# Detectability of fluorescent gold nanoparticles under micro-CT and optical projection tomography imaging

**DOI:** 10.1117/1.JMI.7.2.026002

**Published:** 2020-04-06

**Authors:** Stevo Kozomara, Nancy L. Ford

**Affiliations:** a The University of British Columbia, Department of Oral Biological and Medical Sciences, Vancouver, British Columbia, Canada; b The University of British Columbia, Department of Physics and Astronomy, Vancouver, British Columbia, Canada

**Keywords:** gold nanorods, nanoparticles, melanoma, micro-computed tomography, optical projection tomography

## Abstract

**Purpose:** Preclinical studies often compare micro-computed tomography (micro-CT) imaging with histology using optical microscopy of fluorescently labeled slides. However, correlating the images is difficult because the tissues appear differently in the two modalities. It would be valuable to have a single contrast medium visible on both radiographic and optical imaging.

**Approach:** We have explored the detectability of fluorescently labeled gold nanoparticles under micro-CT and optical projection tomography (OPT) in agarose phantoms and a murine melanoma tumor model. Murine melanoma cells were used to induce tumor growth in the right hind legs of 12 C57Bl6 mice, with the maximal tumor size of $1\;{\mathrm{cm}}^{3}$. We injected Cy3 fluorescently coated gold nanorods directly into the tumors. The mice were scanned with *in vivo* micro-CT (for pre- and post-contrast scans). Once euthanized, the hind leg was dissected and scanned with a higher resolution specimen micro-CT and OPT.

**Results:** The distribution of the gold nanoparticles appeared to be contained and isolated to the tumor. Alignment of micro-CT specimen scans with the OPT scans was possible, although there was also autofluorescence of the surrounding muscle tissue.

**Conclusions:** This study highlights the potential use of fluorescently labeled gold nanoparticles for imaging murine melanoma tumors using micro-CT and OPT.

## 1 Introduction

In small animal imaging for preclinical research, micro-computed tomography (micro-CT) is often the imaging modality of choice, due to its high-spatial resolution, excellent sensitivity to both bone and lung, relatively short scan times, and cost-effectiveness.^1^ However, solid tumors have densities similar to their surrounding soft tissues; therefore, to be able to view them using micro-CT, the use of a contrast agent is required. Tumors are often highly vascular with a vascular architecture that is dysfunctional and leads to the leakage of substances from the vascular system into the surrounding tumor. This is collectively known as the enhanced permeability and retention effect.^2^ Using this characteristic to our advantage, and the fact that particle sizes of 200 to 300 nm can readily extravasate into the tumor tissue, nanoparticle contrast media can be used to visualize tumors on micro-CT scans.^3^^,^^4^

The use of gold nanoparticles as a contrast agent would appear ideal, as gold has a high atomic number and high density, both of which would provide favorable x-ray attenuating properties.^5^ Gold nanoparticles are of particular interest in providing contrast for computed tomography for several reasons, including the potential for long circulation times, the ability for conjugation of various functional groups and coatings to target specific tissues, and its general stability. In addition, gold provides ∼2.7 times greater contrast per unit weight than iodine, can be manufactured in different sizes and shapes, can have its surfaces modified, and is generally considered nontoxic.^5^^,^^6^

While it is possible to use a contrast agent to assist in isolating and identifying tissues in computed tomography scans, these same contrast agents are not visible when attempting to view them under optical imaging. For comparison with micro-CT, a three-dimensional (3-D) optical technique is preferred, such as optical projection tomography (OPT), which reconstructs projection images around the sample similar to CT. OPT was first described by Sharpe et al. in 2002 with filter sets for fluorescent imaging with Alexa 488 and Cy3 and a bright field channel.^7^ Images of mouse embryos, including different phenotypes, were presented in that paper, but the technique was noted to be limited by penetration of the light into thicker tissues and scattering effects from the tissue.^7^

Our hypothesis is that, by using fluorescently labeled gold nanorods injected into mice, we could provide contrast for *in vivo* micro-CT imaging, and fluorescence for optical projection tomography imaging, enabling detection of the gold nanorods in the two imaging modalities. In this research paper, we show that fluorescently labeled gold nanoparticles can provide satisfactory contrast under micro-CT when injected directly into induced murine melanoma tumors. Using Cy3 fluorescently labeled gold nanorods, which will fluoresce at the excitation wavelengths produced by the OPT scanner, provides adequate attenuation required for micro-CT imaging and fluorescence required for visual recognition when using UV filtered light with the OPT scanner.

## 2 Materials

### 2.1 Gold Nanoparticles

The fluorescently labeled gold nanorods (Nanopartz^®^ Inc., Loveland, Colorado) were 10  nm×40  nm, coated with Cy3 fluorophore (555/570  nm), and suspended in a sterile phosphate-buffered saline (PBS) solution. We used two concentrations of nanorods: 50 OD concentration, which had 3.15  mg Au/ml, and 250 OD concentration, which had 12.18  mg Au/ml and is the most concentrated available. Since the gold will settle to the bottom of the tube in time, we agitated the tube for 60 s on a vortex mixer, then centrifuged for 60 s at 900 rpm prior to use. Each time a sample was taken from the tube, the micropipette tip was inserted into the center of the solution and held approximately in the middle of the remaining solution to ensure reproducible results.

### 2.2 Micro-Computed Tomography

All of the *in vivo* scans were done using an *in vivo* micro-CT scanner (eXplore CT 120, TriFoil Imaging, Chatsworth California) reconstructed with 50  μm isotropic resolution. Images were obtained with a step-and-shoot protocol, at 80 kVp, 40 mA, with a total scan time of 8.5 min and an estimated entrance dose of 0.32  Gy/scan.

A specimen micro-CT machine (Scanco Medical μCT100, SCANCO Medical AG, Brüttisellen, Switzerland) was used for all high-resolution scans. For the phantom study, images were acquired with 7.4  μm isotropic resolution. For the tumor study, depending on the size of the sample, 10 to 17  μm isotropic resolution was used. The imaging protocols for the specimen scanner included beam energy at 90 kVp and an intensity of 200  μA, with scan times ranging from 109 to 144 min.

### 2.3 Optical Projection Tomography

The Bioptonics OPT Scanner 3001M (MRC Technology, Edinburgh, Scotland, UK) was used for all OPT imaging. The light source used was filtered UV light at 545 to 610 nm wavelength to correspond with the excitation wavelength of the Cy3 fluorophore on the gold nanoparticles. Samples were adhered to the sample mount using super glue (Instant Adhesive 200, Permabond LLC, Pottstown, Pennsylvania) and submerged in a cuvette containing BABB (1-part benzyl alcohol and 2-parts benzyl benzoate) during the scan. For the phantom study, we used 1000 ms exposure time per projection and reconstructed with 8.5 to 10  μm voxels. For the tumor model, we used an exposure range of 300 to 600 ms per projection and reconstructed with 20  μm voxels.

## 3 Methods

### 3.1 Agarose Phantom

A solution of 1% agarose was prepared using 0.25 g of agarose powder, dissolved in 25 ml of water, and heated using a heating element. Once dissolved, the solution was allowed to cool on a bed of ice for ∼15  min, to the point of having thickened but still remaining relatively fluid. Using a micropipette, we added 2  μl of the gold nanorods into the center of the agarose solution and allowed it to set in the refrigerator at 4°C. Once the agarose had fully set, it was trimmed to $∼1\;{\mathrm{cm}}^{3}$ using a standard shaving razor blade. For each concentration (50 and 250 OD) of nanorods, we prepared two agarose phantoms for analysis.

Each phantom was scanned first using the specimen micro-CT scanner, as described in Sec. 2.2. Then the agarose phantom was cleared in 100% methanol (30 ml) for a total soak time of 43 h. The methanol was replaced with fresh methanol, at time points of 2, 6, and 19 h. Then the phantom was submerged in BABB for an additional 24 h. The agarose phantom was then removed from the BABB and adhered to the specimen mount for OPT scanning, as described in Sec. 2.3.

### 3.2 Animal Model

Ethics approval (A14-0125) was obtained prior to commencing with the small animal research. Twelve female nine-week-old C57Bl6 mice were purchased from Charles River (Wilmington, Massachusetts) and housed with a standard day and night cycle. The mice were fed a standard mouse diet with free access to food and water throughout the study.

B16-F10 cells were purchased from ATCC^®^ (CRL-6475™, American Type Culture Collection, Manassas, Virginia) and were grown in uncoated cell culture-grade dishes in Dulbecco’s modified Eagle medium (DMEM, Cat#: ATCC-302002) containing 10% fetal bovine serum and penicillin–streptomycin (Fetal Bovine Serum, Cat#: 12484-010, Gibco™, Merck KGaA, Darmstadt, Germany).

Prior to injection, the culture medium was removed, and the cell layer was rinsed with PBS (Cat#: 20012-027, Gibco™, Merck KGaA, Darmstadt, Germany). We then added 1.0 ml 0.25% (w/v) trypsin-EDTA solution (Cat#: 25200-056, Gibco™, Merck KGaA, Darmstadt, Germany) to detach the cells from the substrate. Cells were washed with PBS and cell numbers were counted using a hemocytometer. After spinning at ∼125×G for 5 min, B16-F10 cells were resuspended in PBS at a concentration of $3\times 10^{5}\;\text{cells}/\mathrm{ml}$. Under inhaled anesthesia (2% isoflurane in $O_{2}$), 0.1 ml of the cell solution was injected subcutaneously into the right hind leg of each mouse, approximately at the midshaft of the femur. The mice were returned to the animal facility for approximately 3 weeks, until the tumors reached a maximum size of about $1\;{\mathrm{cm}}^{3}$, as measured with calipers.

Once tumor growth had completed, each mouse received pre- and post-contrast micro-CT scans. Anesthesia was induced with 5% isoflurane in $O_{2}$, and the mouse was positioned prone on the scanner bed where a nose cone was placed to maintain anesthesia using 1.5% to 2% isoflurane in $O_{2}$. Precontrast scans were performed as described in Sec. 2.2. Without moving the mouse, the tumors were directly injected with 20  μl of 250 OD Nanopartz^®^ gold nanorods using a 1-ml insulin needle of 29 gauge. When the injection of gold nanorods was completed, the needle was left in place for 10 s, to ensure the contrast had time to enter the tumor. A postcontrast scan was then performed.

The mice were then euthanized with inhaled 100% carbon dioxide (${\mathrm{CO}}_{2}$). Once euthanized, the fur of the right hind leg was removed with a commercially available hair removal product (Nair^®^), and the hind leg was dissected and placed in 40 ml of 10% buffered formalin for 2 days, after which it was scanned with the specimen micro-CT scanner as described in Sec. 2.2.

Once the specimen scan was complete, each hind leg was placed in 40 ml of 100% methanol, which was replaced daily for 4 days, after which it was placed in 40 ml of BABB for 2 days. Once cleared, the samples were then placed into 1% agarose, which was allowed to cool and set in the refrigerator, after which it was trimmed to ensure it would fit in the OPT cuvette. The agarose-embedded right hind leg was placed once again into 40 ml of 100% methanol for 4 days (the methanol was replaced daily) after which it was placed into 40 ml of BABB for 2 days. This was done to clear the agarose surrounding the sample. The samples were then scanned with the OPT scanner as described in Sec. 2.3.

### 3.3 Measurements and Analysis

MicroView (Version ABA 2.2, Trifoil Imaging, Chatsworth, California) was used to view the pre- and post-contrast *in vivo* micro-CT scans. The pre- and post-contrast *in vivo* scans were registered using six reference points from the right hind leg of each mouse using a rigid-body registration algorithm. We also assessed and viewed the specimen micro-CT scans and OPT scans, using MicroView, with additional visualization of the scans using Amira (Version 6.0.1, Thermo Fisher Scientific, Waltham, Massachusetts).

The tumors were measured clinically with digital calipers. The clinically measured tumor size was approximated using the calipers, but as the tumors were invasive and penetrated the soft tissue, a third measurement to determine depth was not possible. However, using Eq. (1), we can estimate the volume (V) of the tumors from the measured length (L) and width (W).^8^

$$V=\frac{W^{2}L}{2}.$$ (1)

Tumor volumes were measured in the *in vivo* micro-CT images using the advanced region of interest feature in MicroView. The software allows the user to browse through the slices of the micro-CT scan and manually outline the margins of the tumor. Then a 3-D volume representing the tumor can be created and the volume recorded.

## 4 Results

### 4.1 Agarose Phantom

**Fig. 1:**
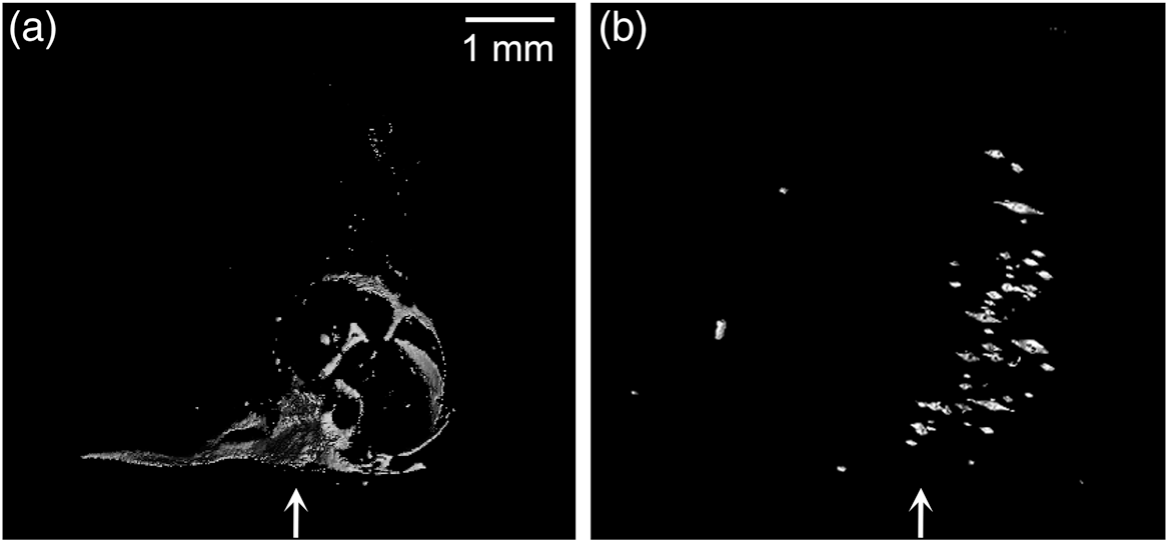
Surface rendering of the 250 OD gold nanorods in the agarose phantom showing (a) regions with high attenuation in micro-CT at 7.4  μm resolution and (b) fluorescence signal with OPT at 8.65  μm resolution. Arrows show approximate location of the injection site for the nanorods.

The agarose phantom results showed visibility of the gold with high-resolution micro-CT and fluorescent signal with OPT, as shown in Fig. 1. It was difficult to align the individual particles exactly, but localization of the enhanced regions was achieved. From these results, we determined that introducing the nanoparticles into a small, well-defined area would enable detection with both x-ray and optical modalities.

### 4.2 Animal Model

**Fig. 2:**
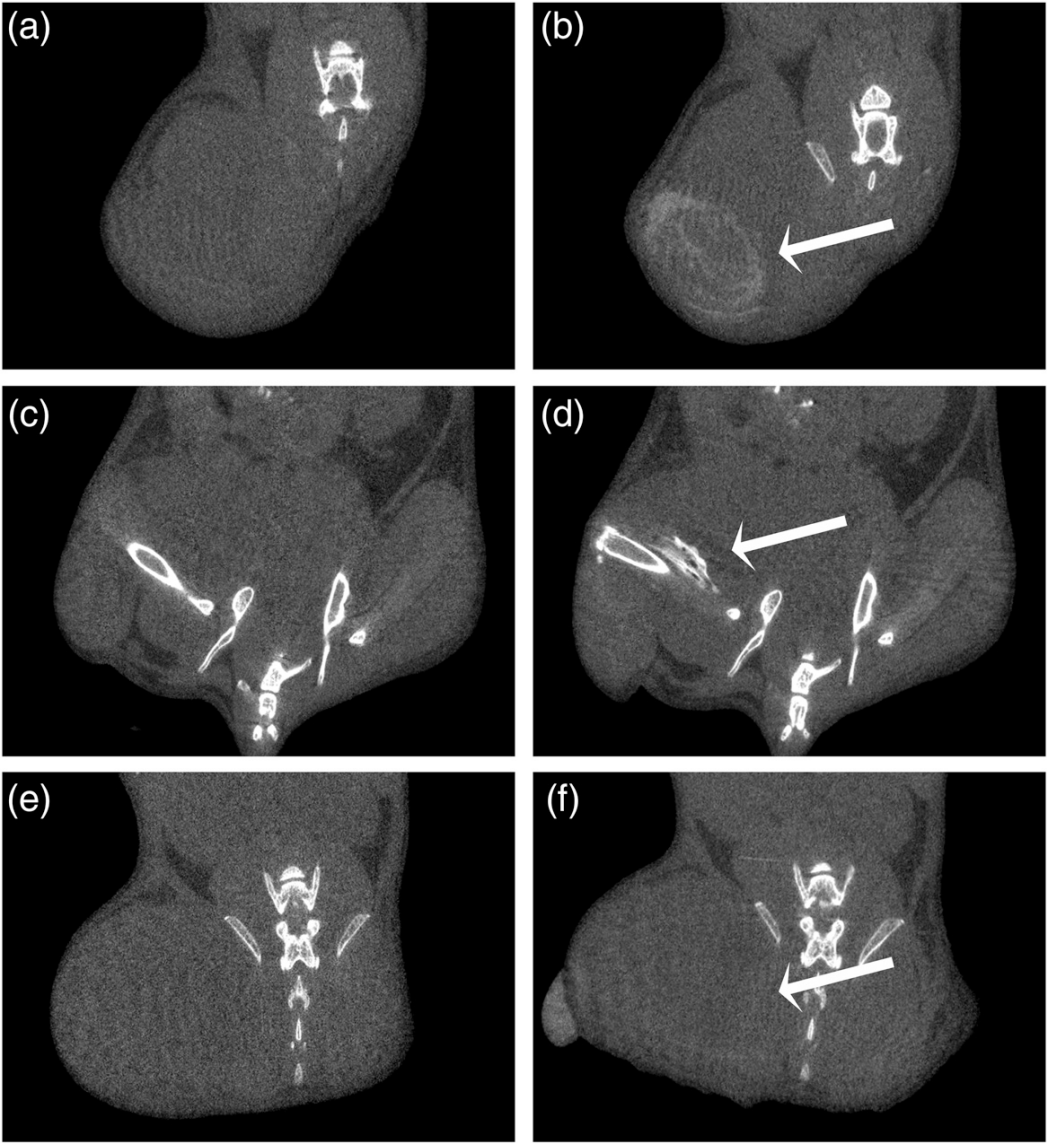
Images of gold nanorods providing contrast for *in vivo* micro-CT scan at 50  μm resolution. (a), (c), and (e) pre-contrast and (b), (d), and (f) post-contrast injection of 20  μl of gold nanorod solution of mouse #7 [(a) and (b)], #9 [(c) and (d)], and #5 [(e) and (f)]. Arrows point to tumor.

**Fig. 3:**
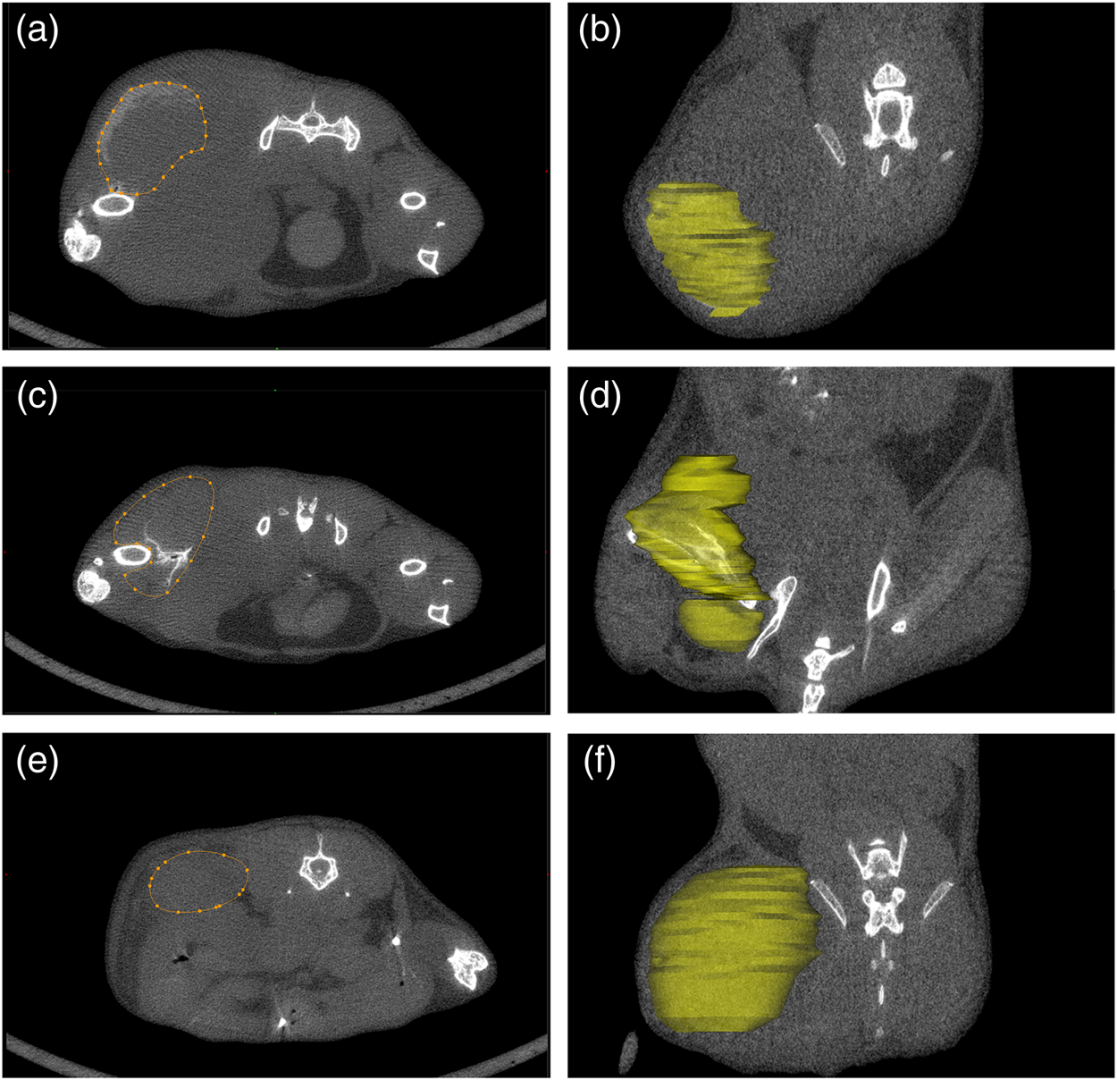
(a), (c), and (e) The contours selecting the tumor, and (b), (d), and (f) the 3-D volumetric region of interest for mouse #7 [(a) and (b)], #9 [(c) and (d)], and #5 [(e) and (f)].

**Fig. 4:**
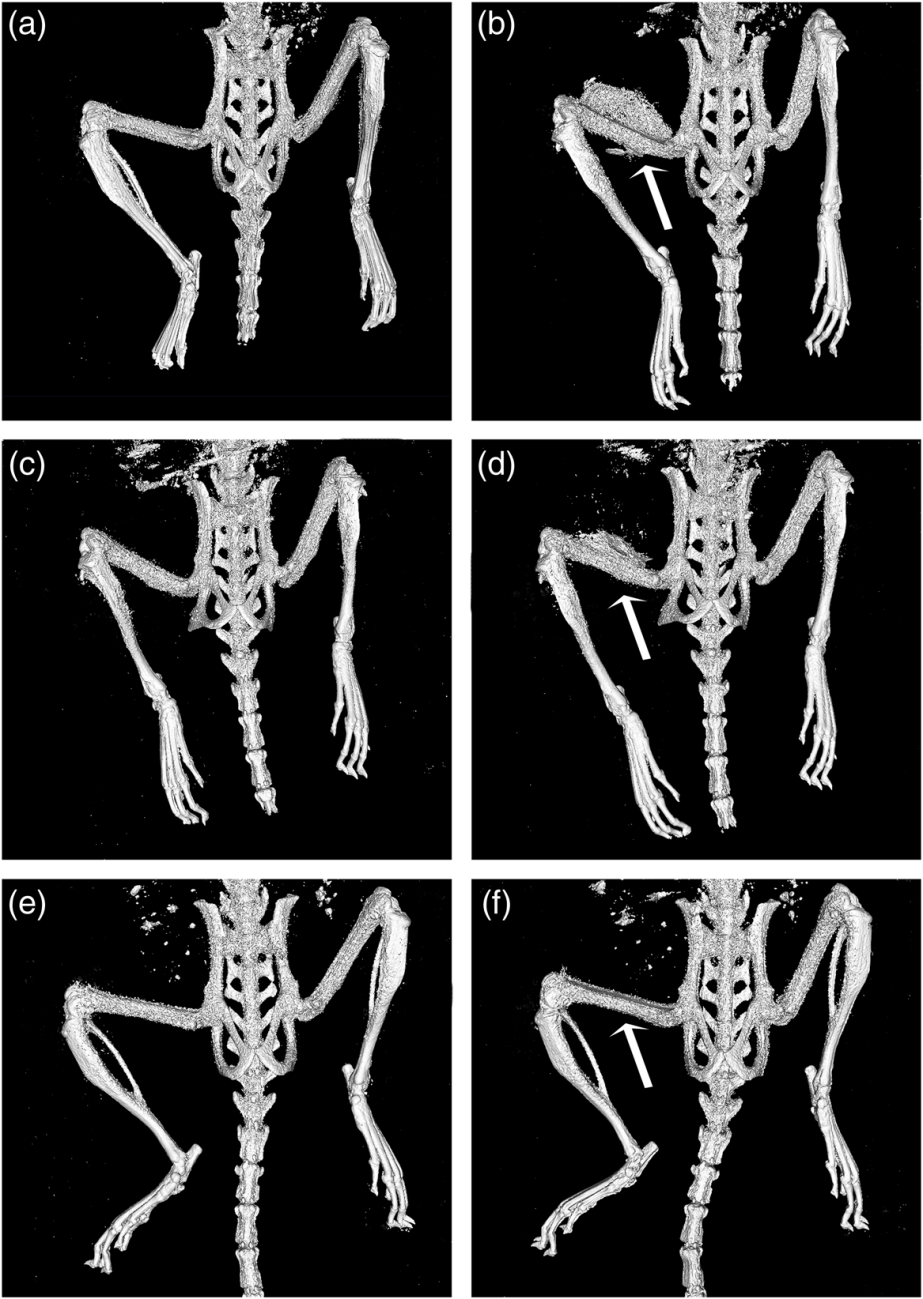
Surface renderings of the bone and gold nanoparticles. (a), (c), and (e) Pre-contrast and (b), (d), and (f) post-contrast injection of mouse #7 [(a) and (b)], #9 [(c) and (d)], and #5 [(e) and (f)]. Arrows point to gold nanoparticle accumulation at the tumor site. Additional high-density regions present near the top of each pair of images are due to material in the intestinal tract.

The tumor could be easily visualized in the postcontrast *in vivo* micro-CT scans as shown in Fig. 2. Figure 3 shows the tumor segmentation procedure, including the manual contours on a selected axial view and the 3-D region of interest resulting from the segmentation. From the 3-D regions of interest, we calculated the corresponding tumor volume. There was clearly some variability in the measured tumor volumes between the mice and between what we measured using software and estimated clinically. Some of the tumors that were measured as being quite large using the software were estimated to be much smaller using the calculated volumes from our caliper measurements. Although the mice were monitored daily and their tumor size assessed using calipers, the actual tumor sizes themselves varied considerably between the mice at the point of euthanasia. In particular, mice #1 to 3 had tumors that were much larger than the remaining mice, as shown in Table 1, which was due to the fact that these mice were allowed to grow their tumors for 3 weeks. One additional mouse (mouse #8) also exhibited larger tumors than the other mice due to the tumor growing into the muscle, which was difficult to assess in awake mice with calipers. The remaining mice were monitored until the sizes of the tumors dictated their experimental end point, as shown in Table 2. It should be noted that we were unable to visualize and assess the tumor found in mouse #11, both pre- and post-contrast administration. From the *in vivo* micro-CT scans, it was noted that the contrast agent was not in fact present in the tumor itself but appeared to have leaked out of the injection site and deposited in the fur of the mouse instead. This was verified because, on the *in vivo* scans, the contrast can be seen on the outer most surface (fur) of the right hind leg, while when viewing the specimen scan (which was taken after the fur was removed), the contrast was no longer visible. For the remaining mice, the *in vivo* pre- and post-contrast scans, once registered, allowed for comparison and provided evidence that indeed contrast was present. With nothing other than contrast agent being introduced into the mouse between the scans, the resulting radiopacity must be the result of the injected gold nanoparticles. Figure 4 shows isosurface renderings of the bone and gold nanoparticles for three mice, demonstrating varied results, from very little or no contrast being evident in some scans to being able to differentiate the tumor very well from the surrounding tissues in other scans.

**Table 1:** Measured tumor volumes for large tumors ($\text{tumors}>1\;{\mathrm{cm}}^{3}$); caliper measurements given as dimensions in column 3 and used to estimate the volume using Eq. (1) in column 4; image-based volume from the postcontrast *in vivo* micro-CT scan in column 5.

Mouse	Weight (g)	Clinically measured tumor dimensions (mm)	Estimated tumor volume (${\mathrm{mm}}^{3}$)	Image-based tumor volume (${\mathrm{mm}}^{3}$)
1	25.6	13×13	1099	7575.6
2	23.2	12×12	864	2910.7
3	23.8	11×11	665	2555.5
8	21.8	10×10	500	1099.0

**Table 2:** Measured tumor volumes for smaller tumors ($\text{tumors}\leq 1\;{\mathrm{cm}}^{3}$); caliper measurements given as dimensions in column 3 and used to estimate the volume using Eq. (1) in column 4; image-based volume from the postcontrast *in vivo* micro-CT scan in column 5.

Mouse	Weight (g)	Clinically measured tumor dimensions (mm)	Estimated tumor volume (${\mathrm{mm}}^{3}$)	Image-based tumor volume (${\mathrm{mm}}^{3}$)
4	19.0	10×10	500	605.0
5	18.7	10×10	500	859.0
6	19.1	9×9	365	506.8
7	18.2	9×9	365	436.4
9	20.6	8×8	171	404.4
10	20.4	9×9	365	837.9
11	20.6	8×8	171	Could not assess
12	21.3	8×8	171	219.0

**Fig. 5:**
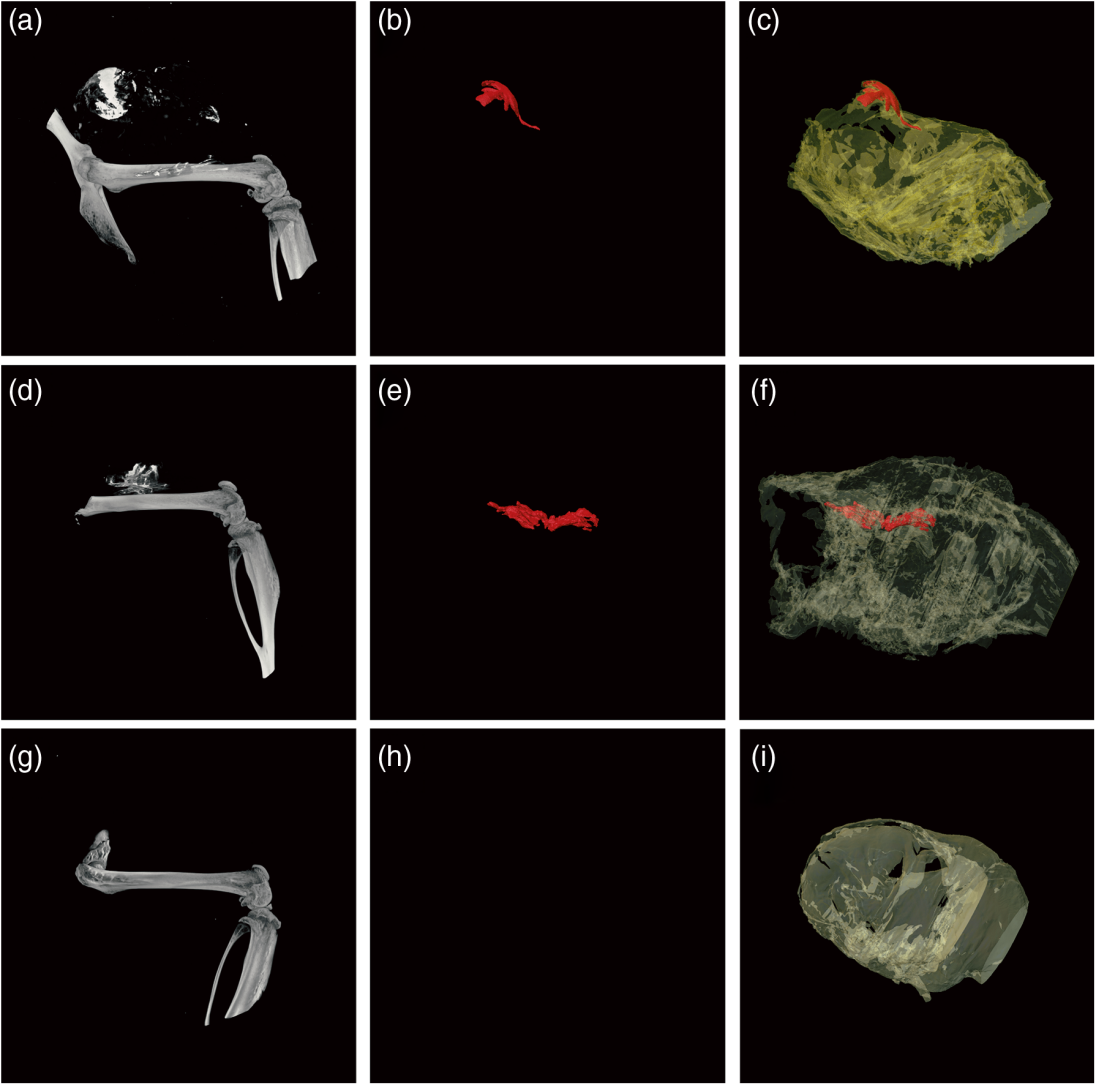
Surface renderings of the bone and gold from (a), (d), and (g) micro-CT and (b), (e), and (h) OPT images showing fluorescence, and (c), (f), and (i) with the soft tissue shown (transparent) of mouse #7 (top), #9 (middle), and #5 (bottom).

Figure 5 shows surface renderings from the specimen micro-CT scans of the excised hind legs in (a), (d), and (g), with the corresponding OPT images showing the fluorescent signal in (b), (e), and (h) and with the tissue shown in transparent color in (c), (f), and (i). The specimen scans provided more favorable images when visualizing the contrast agent as these were taken at a higher resolution, ranging from 10  μm for the smaller tumors and 17.2  μm for the larger tumors. By increasing the resolution of the scan, we effectively improve the spatial and contrast resolution compared with the *in vivo* images, allowing for improved visualization and differentiation between the tumor and surrounding tissues.

The OPT scans proved more challenging, and while the images did show fluorescence that appeared to correspond to the contrast seen in the micro-CT scans, there was an abundance of background autofluorescence from the surrounding soft tissues. While the gold particles may have been detected in the OPT images, the abundance of background fluorescence and signal made it difficult to isolate the fluorescently labeled gold nanoparticles or to co-localize with the micro-CT images.

## 5 Discussion

The fluorescently labeled gold nanoparticles employed in this research were intended for use in either two-dimensional (2-D) confocal or fluorescent microscopy. Our study is the first to use these fluorescently labeled nanoparticles as contrast media for micro-CT imaging and the first attempt to co-localize the particles under two different imaging modalities (optical and x-ray). The Cy3-coated gold nanorods could be visualized in the images from *in vivo* and postmortem micro-CT and OPT, and the tumor locations in the postmortem images were successfully co-localized, mainly using the micro-CT images for tumor delineation.

An advantage to the use of gold nanoparticles as a preclinical contrast agent is that it allows for a single bolus injection, without the need for constant rate infusion. Depending on the size and shape of the particles, including the coatings or functional groups used, long circulation times are possible.^5^ Additionally, gold as a whole is considered nontoxic and biocompatible and can be used to provide adequate contrast in micro-CT when injected directly into tumors.^9^ Several studies have looked at gold nanoparticles (both as spheres and rods) as an alternative to iodine as a preclinical CT contrast agent, and the attenuation reported has varied from 2.7 to 5.7 times that of iodine.^6^^,^^10^ While it is known that gold nanoparticles can provide attenuation, very few studies have assessed the effect of the size of the gold nanoparticles on attenuation. Xu et al.^11^ used gold nanoparticles of varying size and concluded that the smaller gold nanoparticles exhibited greater x-ray attenuation; however, the contrast enhancement may come at a cost of toxicity effects for the mouse.^12^

A major benefit of gold nanoparticles is the opportunity to target specific locations or receptors within the body. *In vivo* targeted delivery of bisphosphonate-functionalized gold nanoparticles as contrast for detection of breast cancer calcifications that would have gone unnoticed under standard mammography imaging has been reported.^13^ Popovtzer et al.^14^ demonstrated that, using gold nanoparticles conjugated to UM-A9 antibodies, the gold nanoparticles could accumulate in the targeted cells at a rate 5 times higher than using nontargeted gold nanoparticles or no contrast at all. By utilizing the characteristic overexpression of transferrin on tumor cells, gold nanoparticles with an antibody for transferrin could be used to target these cells specifically,^15^ allowing for drug delivery systems to target tumor cells specifically, or used as a radiosensitizer for elevated radiation dose enhancement.^16^ Hainfeld et al. studied the effect of gold nanoparticles injected intravenously *in vivo* using mice with terminal intracerebral gliomas. The results found that the tumors had taken up the gold nanoparticles at a rate of 19:1 versus normal brain tissue and that the resulting increase in irradiation dose was 300% higher, leading to a 50% > 1-year survival rate, compared with controls that had a 100% mortality rate.^17^ Other researchers have conjugated gold nanoparticles to carbohydrates, including glucose, mannose, and galactose, to target various tissues and study carbohydrate metabolic processes,^18^ while the conjugation of gold nanoparticles to TAT peptides (which are cell-penetrating peptides that facilitate the transfer of molecules across the cell) has also been studied, allowing specific delivery of pharmaceutical payloads in the study of HIV.^19^ The possibilities appear endless as the ability to conjugate virtually any functional group, including fluorophores, to gold nanoparticles, plus their characteristic small size, makes them virtually perfect as a vector.

However, challenges remain in using fluorescently labeled gold nanoparticles, including determining the concentration of particles needed to achieve adequate contrast, while not using more product than is necessary and ensuring the contrast agent is well-distributed to enable clear delineation of the tumor margins. We ran into issues with being able to standardize the size of the initial melanoma tumors. It has been reported previously that the B16-F10 murine melanoma strain grows with relatively large variability in tumor volume.^20^^–^^22^ Adding to the difficulties of working with live mice, being able to accurately measure an invasive tumor clinically using calipers was more of a challenge than initially anticipated, and some of the initial tumors were very large as compared with latter ones. The caliper measurements were not able to accurately detect the tumor depth, leading to substantial discrepancies between the image-based measurements of tumor volume and those calculated using the caliper measurements. In some of the mice, the tumors penetrated deeper into the muscle, which would go unmeasured by the caliper method.

By performing surface rendering in the *in vivo* images, we were able to clearly identify the tumor periphery in several mice (Fig. 4). The oval or circular shape of the melanoma tumor is common in the early growth stages of soft tissue tumors, and the contrast present in the *in vivo* scans correlated well with the higher resolution scans from the specimen scanner. However, a curious finding was in the dispersion of the gold nanoparticles. Some of the mice clearly showed a more uniform and rapid dispersion of contrast, while in others it seemed to pool and localize at the site of injection. It is possible that if we would have allowed additional time between the injection and the *in vivo* micro-CT scan, a more uniform dispersion of the nanoparticles may have been achieved.^2^ Tumors, particularly benign but also metastatic tumors in the early stages, are surrounded by a fibrous, connective tissue capsule that retains the tumor cells, which may also retain the gold nanoparticles.^23^^,^^24^ The distribution of the particles, along with the limitations of the penetration of light into tissue, meant that the tumors were much easier to delineate in the micro-CT images than in the OPT images. Other researchers have recently reported their methods of simultaneous imaging using a micro-CT scanner with an added optical sensor to detect nanocrystals developed in their lab in a mouse tumor model.^25^ Using simultaneous image acquisition, the anatomy was registered between the modalities, but the tumor location was only well-defined in the micro-CT images, similar to our results, due to limitations in optical imaging of tissues, such as scatter and limited penetration depth.^25^ These researchers suggest that their simultaneous acquisition approach represents an improvement over sequential acquisitions for the different modalities by eliminating movement between the images.^25^

The main purpose of this paper was to be able to detect the fluorescent gold nanoparticles in the images obtained from micro-CT and OPT. Using a fluorescently labeled contrast agent, additional preparation and/or staining would not be required to view the same contrast agent in OPT as in micro-CT. In the OPT images, the soft tissue surrounding the tumors (muscle tissue fiber) seemed to provide an autofluorescence signal with the same wavelength range as the fluorescent label, which we believe may be related to the fixation process. Biogenic amines will react with formaldehyde to produce compounds with a maximum emission of 480 nm.^26^ Additionally, there have been reports of autofluorescence due specifically to the use of aldehydes as fixatives; to reduce the autofluorescent signal, it has been suggested to use a sodium borohydride bath after fixation, or avoid aldehyde fixatives altogether.^27^ Alternative fixatives include those based on alcohols (mixtures of methanol, ethanol, and propanol) or simply minimizing the percentage of aldehydes in the fixative agent.^28^ While we were able to achieve detection of the gold nanoparticles using both micro-CT and OPT imaging, it was clear that the autofluorescence of the muscle tissue interfered with the interpretation and correlation of the results.^29^ For future studies, it may be advantageous to consider using different fluorescent labels on the gold nanoparticles, which may enable better separation of the nanoparticle fluorescence signal from the background tissue autofluorescence. In addition, an alcohol-based fixation method would also be beneficial.

## 6 Conclusions

The use of a Cy3 fluorescently labeled gold nanorod contrast agent to visualize tumors using both micro-CT and OPT imaging proved successful. The innovation of this project was the use of a Cy3 fluorescently labeled gold nanoparticle, which was intended for 2-D microscopy, to provide contrast in 3-D x-ray imaging. We demonstrated that the gold particles were observed in both the micro-CT and OPT images using an agarose phantom. Using a tumor model, we could use *in vivo* micro-CT imaging to visualize the gold nanoparticles, while using the specimen scanner allowed for a much better and higher resolution image, detailing exactly where the contrast agent was present in the excised hind leg. While OPT presented with its own set of challenges, the images did provide some level of fluorescence, and we were able to detect the tumor in both the OPT and micro-CT images.
